# The Central Role of Ribosomal Proteins in p53 Regulation

**DOI:** 10.3390/cancers17101597

**Published:** 2025-05-08

**Authors:** Mikael S. Lindström

**Affiliations:** Department of Medical Biochemistry and Biophysics, Division of Genome Biology, Science for Life Laboratory, Karolinska Institutet, SE-171 21 Stockholm, Sweden; mikael.lindstrom@ki.se

**Keywords:** ribosomal protein, p53, RPL22, MDM2 inhibitors, MDM4, alternative splicing, ribosome biogenesis, chemotherapy resistance, ribosomal stress response

## Abstract

The tumor suppressor protein p53 prevents the malignant transformation of cells by responding to DNA damage, oncogene activation, and abnormal growth signals including ribosome assembly defects. Under normal conditions, p53 activity is controlled by the regulatory proteins MDM2 and MDM4, which suppress its function through ubiquitin-mediated degradation and transcriptional inhibition. A subset of ribosomal proteins initiates the p53 response to impaired ribosome biogenesis. The ability of some ribosomal proteins to control MDM2 and MDM4 activities, and thereby p53, underscores an intriguing aspect of cell biology: proteins primarily known for their roles in ribosome function can exert extra-ribosomal functions. One notable example is the cellular RNA-protein complex involving RPL5, RPL11, and 5S rRNA (5S RNP) which inhibits MDM2 and stabilizes p53. Another RP, *RPL22,* is frequently mutated in cancers with microsatellite instability and its paralog *RPL22L1* is often amplified. Recent studies have revealed that RPL22 directly modulates the alternative splicing of *MDM4* to promote p53 activation, suggesting that the ribosomal protein-p53 relationship is more complex than previously thought. Cellular responses to ribosome biogenesis inhibition extend beyond general alterations in transcription and translation to actively determine cancer cell fate by selectively engaging tumor-suppressor pathways. RPL22’s effect on *MDM4* and other mRNA splicing events is a striking example. A better understanding of the mechanisms involved could guide the development of improved cancer treatments.

## Simple Summary

Ribosomal proteins are essential components of the ribosome known for their role in protein synthesis. However, several ribosomal proteins function outside the ribosome and influence the activity of the p53 tumor suppressor protein. The intracellular RPL5-RPL11-5S rRNA complex blocks the MDM2-mediated degradation of p53. Whether ribosomal proteins inhibit MDM4, which suppresses p53 transcriptional activity, has remained less clear. Several recent studies elegantly demonstrate that RPL22 controls *MDM4* pre-mRNA splicing to boost p53 activity, revealing an additional layer of p53 regulation. *RPL22* is frequently mutated in certain cancer types. Ribosomal protein-mediated control of MDM2 and MDM4 has implications for how cancer cells respond to chemotherapy.

## 1. Introduction

The ribosome, composed of ribosomal RNA (rRNA) and ribosomal proteins (RPs), is essential for protein synthesis and cell growth. Mounting evidence reveals that RPs have functions beyond the ribosome and mRNA translation [1]. Somatic mutations and deletions affecting RPs, such as *RPL5*, *RPL10*, *RPL22*, and *RPS15*, have been identified across multiple cancer types [2,3,4,5,6,7]. Alterations in other RPs have been observed as well [8,9]. These alterations are likely to be involved in tumorigenesis through several mechanisms, including changes in mRNA translation, inactivation of tumor suppressors, increased oxidative stress, and genome instability [10]. A subset of RPs operates in the p53 tumor suppressor pathway, e.g., through the 5S ribonucleoprotein complex (5S RNP), which restrains MDM2-mediated p53 degradation [11]. Recent studies show that RPL22 (eL22) extends this network by affecting the splicing of *MDM4* (*MDMX*), thus modulating p53 through a different mechanism [12]. This commentary discusses the emerging dual roles of RPs in ribosome biogenesis and tumor suppression, with a focus on RPL22’s role in the splicing of *MDM4* [5,13,14]. Understanding how RPL22 controls p53 may have future implications for cancer prognosis and therapy.

## 2. The p53—Ribosome Connection

**Figure 1:**
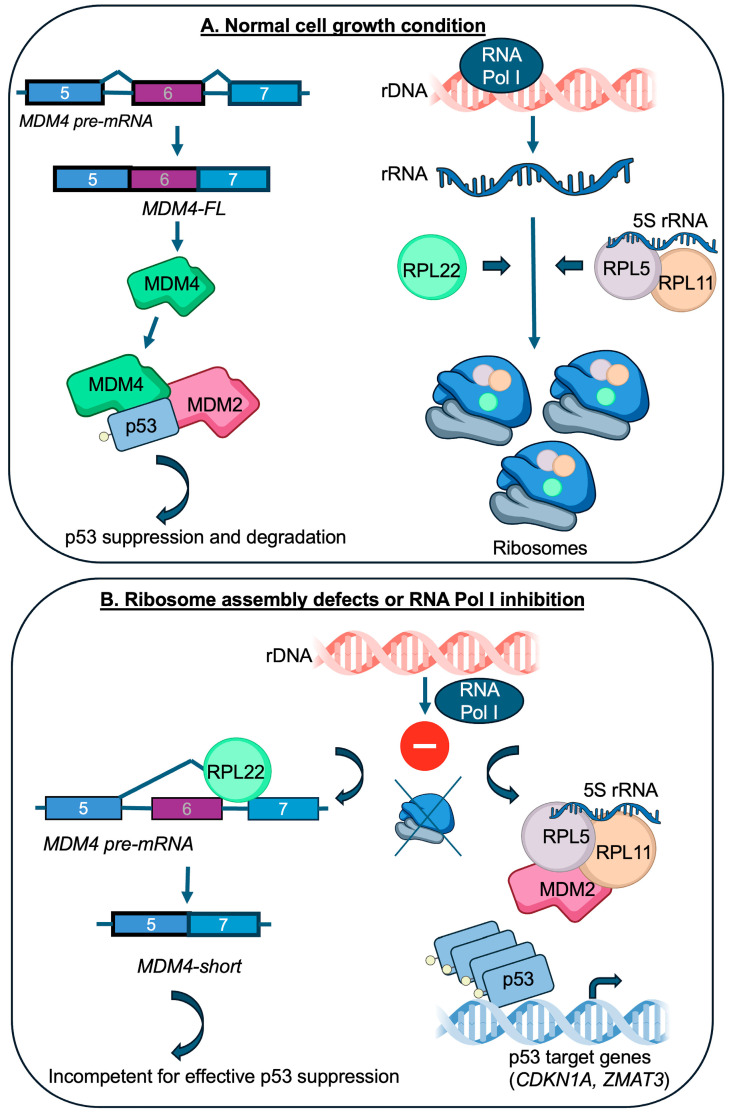
Control of p53 by ribosomal proteins. Under normal cell growth, MDM2 and MDM4 suppress p53 activity, and ribosome biogenesis is ongoing with the incorporation of RPL22, RPL5, and RPL11 into maturing ribosomes (upper panel **A**). In the case of the inhibition or disruption of ribosome biogenesis (RNA Pol I inhibition), RPL22 increasingly binds *MDM4* pre-mRNA, preventing the inclusion of exon 6. This produces a short form of MDM4 that fails to inhibit p53 (lower panel **B**). At the same time, RPL5 and RPL11 together with 5S rRNA tethers MDM2 to prevent it from targeting p53 for degradation. For simplicity, additional proteins associated with the 5S RNP complex are not shown. A few items in the figure are from the NIAID NIH BIOART source available online: https://bioart.niaid.nih.gov (accessed on 4 May 2025). This include item numbers 123, 449, 452, 481, and 473.

p53 acts as a guardian of cellular homeostasis by responding to DNA damage, oxidative stress, and impaired ribosome biogenesis [15]. As mentioned, p53 is suppressed by its negative regulators, MDM2 and MDM4; the inactivation of either is embryonically lethal [16,17]. MDM2, an E3 ubiquitin ligase, promotes p53 degradation, while MDM4 inhibits p53’s transcriptional activity [18]. *MDM2* and *MDM4* are often amplified in cancers leading to the abnormal suppression of p53 [19]. The disruption of ribosome biogenesis is one of the most potent triggers of the p53 pathway [20,21,22,23]. This is thought to be mediated at least in part by the 5S RNP complex, composed of RPL5 (uL18), RPL11 (uL5), and 5S rRNA, a precursor in ribosome assembly within the nucleus [24]. Under normal cell growth conditions, 5S RNP is assembled into ribosomes, but if the ribosome assembly pathway is dysfunctional, its free form binds to MDM2, blocking its ability to degrade p53, thereby inducing cell cycle arrest (Figure 1) [11,25,26]. The loss of RPL5 or RPL11, two RPs that work in tandem, disables this p53 checkpoint [11,26,27,28]. A recent study provided much-needed biochemical insights into the 5S RNP-MDM2 complex and described a physical association with the SURF2 (Surfeit 2) protein [29]. SURF2 acts as a buffering component within the 5S RNP by antagonizing MDM2. The depletion of SURF2 activates p53 by allowing more MDM2 to be tethered to 5S RNP. Other proteins bound to this complex include HEATR3 and La/Sjögren syndrome type B antigen [29].

Ribosomal protein RPL22 has been suspected to influence p53 function. Earlier studies showed that RPL22 can physically bind to MDM2, inhibiting its ability to degrade p53 [30], and RPL22 was also reported to bind *Trp53* mRNA and negatively regulate its translation [31]. While other RPs, such as RPL26 and RPL23, have similarly been implicated in modulating the MDM2-p53 axis, these mechanisms often involve general effects on MDM2 E3 ligase activity or mRNA translation [32,33]. RPs can also act in p53-independent cellular stress responses [34,35]. In contrast, and as will be discussed below, RPL22 exerts a more specific function by controlling the alternative splicing of *MDM4*, thereby adding a new layer to the regulation of p53 activity.

## 3. RPL22 and RPL22L1 Paralog Pair

While RPs are highly conserved, some have paralogs that in rare cases can compensate for the loss of the main variant [36]. RPL22 and its paralog RPL22L1 (RPL22 Like-1) provide an interesting example of compensation, and in this case, it also extends beyond translation to influence cancer-related signaling pathways. The existence of RP paralogs may allow for the fine-tuning of protein synthesis, adapting to the specific needs of different cell types or various stress conditions [37,38]. In cancer, the illegitimate expression of paralogs may lead to changes in ribosome composition, potentially changing the translation of specific mRNAs affecting cell growth or survival. However, the extent to which paralog expression occurs and its relevance in human cells including cancer cells are subjects of ongoing investigations and debate [10,39,40].

RPL22L1 shares 73% amino acid sequence homology with RPL22. RPL22 is normally incorporated into the 60S large ribosomal subunit but RPL22L1 can substitute for RPL22 in ribosome assembly [41]. This compensatory capacity explains why mice lacking *Rpl22* are viable and exhibit only a mild phenotype—*Rpl22-L1*—which compensates for the loss of *Rpl22* [41]. The compensation mechanism is complex, and this paralog pair has been studied in several organisms including yeast, flies, fish, and mammals [41,42,43,44,45]. In mouse cells, Rpl22 can repress the expression of its own paralog *Rpl22L1* by binding directly to its mRNA, preventing translation through splicing alterations [41]. *RPL22L1* is spliced into two or more variants: RPL22L1a, the predominant stable form and incorporated into ribosomes, and a shorter, truncated form, RPL22L1b, which may perform extra-ribosomal functions [46]. A detailed discussion of the intriguing dynamics of the RPL22-RPL22L1 pair is beyond the scope of this commentary, but for further reading and examples see references [47,48,49].

## 4. RPL22 and RPL22L1 Alterations in Cancer

Frequent *RPL22* mutations were discovered in T-cell acute lymphoblastic leukemia (T-ALL) [50], gastric cancer [51], and endometrial cancer as reported at the end of 2012 [52]. This coincided with the identification of somatic mutations in *RPL5* and *RPL10* in T-ALL [53]. *RPL22* is often point-mutated, causing frameshifts or harboring other missense mutations, and is also deleted. Additional cancer types with alterations on *RPL22* include ovarian cancer, adrenocortical carcinomas, hepatocellular carcinomas, and colon adenocarcinomas. See for example, cBioPortal, available online: https://www.cbioportal.org (accessed on 4 May 2025). Mutations and the downregulation of RPL22 expression have been observed in other malignancies as well. *RPL22* point mutations are especially prevalent in cancers classified as microsatellite instability-high (MSI-H) [5]. These cancers often display the point-mutated allele *RPL22 p.K15fs*, explained by the fact that *RPL22* has vulnerable coding mononucleotide repeats. At the time these mutations were initially reported, the functional and clinical significance was not clear, however, a tumor-suppressive role in T-cell lymphoma was suggested [50]. The correlation between mutant *RPL22*, *RPL22L1*, and *MDM4* splicing pattern was described a few years later [54]. *RPL22L1* expression is typically low in normal tissues but is upregulated in response to *RPL22* loss of function. Moreover, *RPL22L1* is frequently amplified in certain cancer types. See for example, cBioPortal, available online: https://www.cbioportal.org (accessed on 4 May 2025).

## 5. RPL22 Becomes Connected to MDM4 and p53

Splicing is an important mechanism affecting MDM4 function [19,55]. The inclusion of exon 6 results in the production of full-length MDM4, which effectively suppresses p53. In contrast, the exclusion of exon 6 produces a shorter, unstable MDM4 isoform that fails to inhibit p53 effectively [55]. The splicing of *MDM4* is governed by multiple proteins, including the p53 target ZMAT3 (previously known as Wig-1), which promotes exon 6 skipping [56]. In 2023 and 2024, several groups published exciting findings that clarified the functional link between RPL22 and MDM4 (see timeline in Figure 1). As a prelude, Howard et al. (2023) provided a ribosome-centered angle on MDM4 and p53 signaling [13]. Their lab studied inhibitors targeting the chromatin-associated protein WDR5 (WD repeat domain 5). Interesting findings on their own, inhibiting WDR5 disrupts RP gene transcription, causing ribosome biogenesis stress and the activation of p53 in leukemia cells [13]. Treatment with the WDR5 inhibitors led to changes in *MDM4* splicing (exclusion of exon 6) correlated with reduced levels of RPL22L1 [13].

Weinstein et al., (2024), identified *RPL22* as a tumor suppressor in MSI-H cancers and demonstrated that it alters *MDM4* splicing by directly binding to its pre-mRNA. They also showed that RPL22 controls the splicing of other pre-mRNAs, including *RPL22L1* and *UBAP2L* (Ubiquitin-Associated Protein 2 Like) resulting in decreased levels [5]. The deletion of *RPL22* led to increased inclusion of *MDM4* exon 6, augmenting full length and active MDM4 protein expression. Furthermore, reduced expression of RPL22 was associated with increased proliferation of cancer cells, and resistance to Nutlin-3a, an MDM2 inhibitor. In the same issue, Jansen et al., (2024), elegantly dissected the full mechanism by which RPL22 regulates *MDM4* splicing to activate p53 [14]. This team could show how RPL22 binds to specific elements, stem-loop structures, within *MDM4* intron 6, promoting exon 6 skipping, which then leads to the predominant production of the unstable *MDM4* isoform enhancing p53 activation in response to 5-fluorouracil (a cytostatic compound inducing nucleolar stress). Jansen et al. also described *UBAP2L* and *RPL22L1* splicing by RPL22. Importantly, Weinstein et al., (2024), and Jansen et al., (2024), confirmed the direct binding of RPL22 to *MDM4* pre-mRNA using cross-linking and immunoprecipitation [5,14]. Fan et al., (2024), pre-print, also demonstrated that RPL22 interacts with mRNA splice junctions, affecting the splicing of *RPL22L1* and *MDM4* following RNA Polymerase I (RNA Pol I) inhibition [57]. The study indicates numerous (hundreds) potential splicing changes in response to the targeting of RNA Pol I, suggesting effects on splicing beyond *RPL22L1* and *MDM4*.

Collectively, these studies establish RPL22 as a novel regulator of *MDM4* with possible future implications for cancer prognosis and therapy. They also pinpoint RPL22L1 as a compensatory paralog whose expression increases when RPL22 expression is decreased. It should be noted that the studies mostly rely on cancer cell lines and knockout cell line models in vitro, which may not fully capture the complexity in vivo. While RPL22L1 can partially compensate for RPL22, altered expression level of RPL22 may result in changes in other RPs, including RPL5 and RPL11, because the synthesis of RPs is regulated in a coordinated and balanced manner [58,59]. RPL22’s role in *MDM4* splicing is now clearer, but how it coordinates with other splicing factors is still poorly understood, although there are some clues. SRSF1 splicing factor has already been linked to RPL5 and 5S RNP [60]. Another example is SRSF3 that is connected to *MDM4* splicing [61], and SRSF4 to the splicing of *RPL22L1* [46]. Current evidence presented does not support a direct role for RPL22 in regulating *MDM2* or *TP53* splicing, and it remains to be seen whether there are effects on splicing of p53 target genes.

In summary, the disruption of ribosome biogenesis (inhibition of RNA Pol I) sets free more RPL22 in the nucleus that becomes available to bind *MDM4* pre-mRNA. This is likely to occur in parallel with an increase in free RPL5 and RPL11 that engage MDM2 as is illustrated in the lower panel of Figure 1. In this setting, RPL11 and RPL5 increase both the stability and activity of the p53 protein by inhibition of MDM2, whereas RPL22 has little effect on stress-induced p53 protein stabilization mainly influencing its transcriptional activity. Future work should take care to separate the consequences of *RPL22* loss of function and exchange with RPL22L1 in the ribosome from its extra-ribosomal roles in splicing. A more general experimental challenge in the field is whether the observed effects of drugs or mutant RPs are due to altered ribosome content, ribosomal stress responses, or extra-ribosomal functions [39].

## 6. RPL22-MDM4 and Implications for Cancer Biology

**Figure 2:**
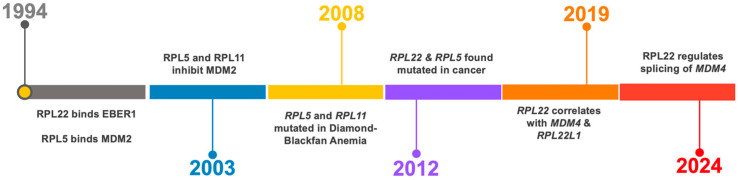
Timeline of discoveries on the role of RPL5 and RPL22 in Diamond–Blackfan anemia [67], cancer [51,52,53], and in the regulation of p53 [5,14,54,68,69,70].

Evidently, RPL22 and RPL5 have now emerged as important regulators of p53 signaling. It is a curiosity that RPL22 and RPL5 were both identified as binding to non-ribosomal targets in the early 1990s and later found mutated in cancers in the 2010s (see a timeline of discoveries in Figure 2). RPL22 was found to associate with Epstein–Barr virus (EBV)-expressed small RNAs (EBERs) [62,63,64,65], while RPL5 was shown to bind MDM2 in association with 5S rRNA [66]. These early findings hinted at extra-ribosomal functions for both proteins, though the biological significance remained unclear at the time. The impact of RP-mediated p53 regulation appears to vary across cancer types and likely has multiple explanations. In some cancers MDM2-mediated p53 degradation may dominate, while others may depend more on the MDM4 suppression of p53 activity [18]. This variation could determine the relative importance of specific RPs in different cancer types. In tumors with mutant p53, RP-mediated control may become less relevant, although keep in mind that MDM2 and MDM4 possess p53-independent functions.

The discovery of the RPL22-MDM4 connection may help resolve several observations in the field that have been difficult to fit within the model of RP-MDM2 dynamics. First, it was observed that MDM4 levels decreased following the inhibition of ribosome biogenesis [71]. That RPL22 directly controls *MDM4* pre-mRNA splicing [12] now emerges as a likely explanation. Second, the activation of the p53-p21 link or induction of p53-dependent apoptosis has occasionally been observed under conditions of ribosomal stress despite the inactivation of the 5S RNP-p53 control [72,73]. It is tempting to speculate that RPL22-MDM4 may be involved in such situations, but this needs to be experimentally tested. Third, the link to MDM4 may resolve some issues on the complex and essential roles of RPL22 and RPL22L1 seen in the development of B and T cells, and the activation of p53 [47,48]. A disruption in the RPL22:RPL22L1 ratio may impact ribosome homeostasis and p53 via MDM4 in hematopoietic stem cells, B and T cells. Fourth, the binding of EBERs to RPL22 with the subsequent induction of RPL22L1 may have a role in modulating growth patterns during EBV latency [74,75], with implications for EBV-positive Burkitt lymphoma.

While RPL22 is mutated in cancer and clearly implicated in *MDM4* splicing, what is the evidence regarding RPL5 and RPL11 in cancer? *RPL5* heterozygous point mutations or heterozygous deletions, which often show an anti-correlation with *TP53* mutations, are observed in glioblastomas, multiple myelomas, breast cancer, and several other cancer types. This supports the idea that some tumors may selectively bypass 5S RNP-mediated p53 activation by reducing the function and/or expression of RPL5 [7]. In contrast, RPL11 mutations are much less frequent and show no clear correlation with p53 status [7]. Mutations in the MDM2 zinc finger, which disrupt its interaction with the 5S RNP complex, have been very useful in biochemical studies, but are exceedingly rare and unlikely to play a major role in cancer. The scarcity of *MDM2* or *RPL11* mutations that would impair 5S RNP control of MDM2 function suggests that tumors rarely target this pathway directly, aside from RPL5 or p53. However, the regulation of the 5S RNP-MDM2 complex could occur through alternative mechanisms, such as altered levels of SURF2 [29]. Importantly, the deletion of *Rpl11* or inactivation of the Mdm2 zinc finger region accelerates lymphoma development in mouse models [76,77]. Furthermore, a study investigating clonal dynamics in hematopoietic cell colonies from individuals with Schwachman–Diamond syndrome (a ribosome disorder with the activation of p53) found that mutations in *TP53*, *RPL5*, and *RPL22* appeared rather frequently as escape mechanisms to overcome p53-imposed growth arrest [78].

## 7. RPL22-MDM4 and Implications for Cancer Treatment

**Figure 3:**
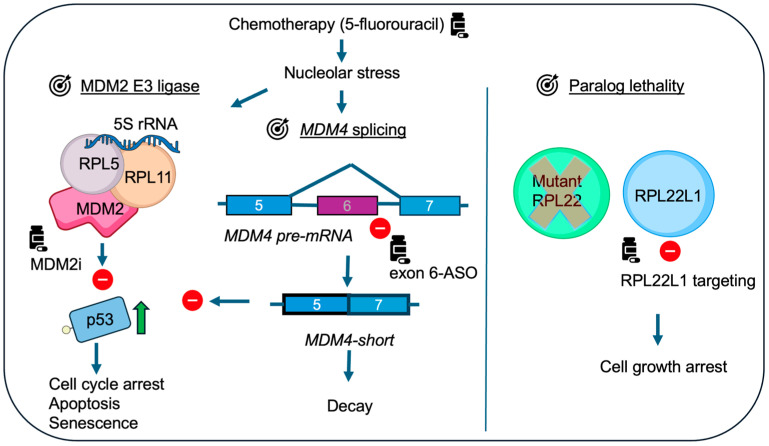
Examples of possible therapeutic strategies in wild-type p53 cancer cells expressing mutant RPL22. Chemotherapy inducing nucleolar stress, e.g., 5-fluorouracil engages the p53 pathway but is less effective due to mutations in RPL22. The inhibition of MDM2 function by MDM2i (e.g., Nutlin-3a) or targeting of *MDM4* splicing to prevent the inclusion of exon 6 can serve to boost p53 activity in this setting (exon 6-ASO: exon 6 antisense-oligonucleotides). Active p53 triggers cell cycle arrest, apoptosis, or other cell fates. The targeting of RPL22L1 may induce growth arrest or lethality in cancer cells regardless of p53 status. A few items in the figure are from the NIAID NIH BIOART source available online: https://bioart.niaid.nih.gov (accessed on 3 May 2025), including item numbers 452, 481, and 473.

One of the key take-home messages from the studies by Weinstein et al., Jansen et al., and Howard et al. is that *RPL22* loss-of-function reduces sensitivity to rather different ribosome biogenesis inhibitors and Nutlin-3a, suggesting a shared putative resistance mechanism across distinct classes of chemotherapies [5,13,14]. RPs including RPL5 and RPL22 influence chemotherapy response in vitro, particularly in cancer cell lines that retain wild-type p53 [5,13,14,79,80]. Given their role in the regulation of p53-MDM2-MDM4, these RPs may affect sensitivity to small molecule MDM2 inhibitors. For example, cancers with reduced *RPL5* might show increased dependence on MDM2 and thus potentially render them more susceptible to MDM2 inhibition. However, the efficacy of MDM2 inhibitors in such context needs to be determined. One also has to keep in mind that alterations in *RPL5* are less frequent, and MDM2 inhibitors are not yet approved for clinical use [18]. Restoring *MDM4* exon skipping, resulting in reduced levels of full-length *MDM4* and enhanced p53 activation, has been suggested as an experimental strategy (Figure 3). Splicing modulators such as SF3B1 inhibitors have been explored in preclinical models to shift splicing patterns in favor of p53 activation, though they can have toxic side effects in normal tissues [81]. In addition to p53-MDM2-MDM4 dynamics, broader alterations in ribosome assembly caused by the loss of *RPL5* or *RPL22*, and general effects on transcription and translation are likely to contribute to chemotherapy responses and resistance.

The roles of RPL22 and RPL22L1 in cancer are of interest beyond their connection to MDM4. Their functional dynamics are likely context-dependent, varying by tissue and cancer type. *RPL22L1* is frequently amplified in cancers, but is this a reflection of a direct oncogenic role (that is increasing MDM4)? It could be argued that the loss of *RPL22* is compensated by RPL22L1 in a way that is not necessarily oncogenic but simply serves to maintain ribosome function. However, the overexpression of RPL22L1 has been associated with enhanced malignant phenotypes in cancer such as increased cell proliferation and resistance to chemotherapeutic agents, such as sorafenib in hepatocellular carcinomas [82], temozolomide in glioblastomas [83], and 5-fluorouracil in colorectal cancer [84]. RPL22L1 expression promotes cell proliferation and invasion, in part, through the ERK signaling pathway [82]. While some of these effects can be potentially explained by unrestrained MDM4 action on p53, findings from studies on alternatively spliced isoforms of *RPL22L1* in glioblastoma [46] suggest the presence of additional more complex mechanisms. Given that the reduction in RPL22 is compensated for by RPL22L1, targeting RPL22L1 may exploit a paralog synthetic lethality approach as indicated [85]. RNA-targeting drugs could be tested experimentally to selectively reduce *RPL22L1* expression (Figure 3). It is important to keep in mind that most RPs are pan-essential and targeting any of them would be expected to result in toxic side effects in normal tissues. But, reliance on paralogs opens an interesting window of opportunity in cancer cells. Finally, RPs are candidates as clinically relevant biomarkers in oncology. RPL22 loss of function correlates with poor prognosis in T-ALL [50,86] and in aggressive microsatellite instability-high (MSI-H) tumors [5], while RPL22L1 overexpression predicts resistance to sorafenib in hepatocellular carcinoma [82]. High RPL22L1 expression in colorectal cancer correlates with poor prognosis [84] and in lung adenocarcinomas [87,88]. These examples underscore the potential of RP-based biomarkers for prognosis, patient stratification, and treatment selection.

## 8. Conclusions

As our understanding of RP biology expands, it becomes increasingly clear that RP mutations and extra-ribosomal RP activities are not merely collateral to tumor progression. First, studies on RPL22 add to a growing body of evidence demonstrating that many RPs have extra-ribosomal functions. Second, RPL22 and RPL22L1 exemplify the complexity of RP paralogs, indicating the broader significance of RP-regulated networks. Third, the role of RPL22 in regulating *MDM4* splicing introduces a new dimension to the p53 pathway, which has traditionally focused on the RP-MDM2 axis. As is now clear, RPL22 mutations disable an important control of MDM4-mediated p53 suppression. The latest studies thus position RPL22 at the center of a ribosome-/nucleolus-related regulatory axis, distinct from yet integrated with, the 5S RNP-MDM2-p53 module. From a therapeutic standpoint, modulating *MDM4* splicing or exploiting RPL22 paralog synthetic lethality may offer strategies for reactivating p53 in cancers with intact but suppressed wild-type function. Even in the context of mutant p53, targeting RPL22L1 could be an option. Several important questions remain: what are the precise mechanisms governing MDM2-5S RNP dynamics, *MDM4* splicing, and *p53* mRNA translation? In addition, there are more fundamental questions: to what extent do these mechanisms drive tumor development versus determine chemotherapy response in vitro and in vivo? And, a question that often comes to mind is why did p53 evolve such an intimate relationship with the ribosome and RPs? A true understanding of these connections may provide insight into cancer development.

## Data Availability

This commentary has not generated new data.
